# Comparison of the impact of type 1 and type 2 diabetes on quality of life of families of patients: A UK cross‐sectional study

**DOI:** 10.1111/dom.16058

**Published:** 2024-11-24

**Authors:** Rubina Shah, Andrew Y. Finlay, Faraz M. Ali, Kennedy Otwombe, Stuart J. Nixon, Lindsay George, Marc Evans, John R. Ingram, Sam Salek

**Affiliations:** ^1^ Division of Infection and Immunity, School of Medicine Cardiff University Cardiff UK; ^2^ Statistics and Data Management Centre, Perinatal HIV Research Unit, Chris Hani Baragwanath Academic Hospital University of the Witwatersrand Johannesburg South Africa; ^3^ Multiple Sclerosis Society Cardiff UK; ^4^ Department of Diabetes University Hospital Llandough Cardiff UK; ^5^ School of Life & Medical Sciences University of Hertfordshire Hatfield UK; ^6^ Institute of Medicines Development Cardiff UK

**Keywords:** diabetes, diabetes secondary burden, family impact, family members/partners, Family Reported Outcome Measure‐16, FROM‐16, quality of life, type 1 diabetes mellitus, type 2 diabetes mellitus

## Abstract

**Aim:**

To measure the impact of type 1 and 2 diabetes mellitus (T1D and T2D) on the QoL of a person's family members/partner and assess if there is any difference in family impact.

**Methods:**

A cross‐sectional study, recruited online through patient support groups, involved UK family members/partners of people with diabetes completing the Family Reported Outcome Measure‐16 (FROM‐16).

**Results:**

Two hundred and sixty‐one family members/partners (mean age = 57.9 years, SD = 13.8; females = 68.2%) of people with diabetes (mean age = 57.7, SD = 20.6; females = 38.3%; T1D *n* = 100; T2D *n* = 161) completed the FROM‐16. The overall FROM‐16 mean score was 10.47, SD = 7.8, suggesting a moderate effect on the QoL of family members of people with diabetes. A quarter (24.5%) of family members experienced a ‘very large effect’ or ‘extremely large effect’ on their QoL. The family impact of T1D (mean FROM‐16 = 12.61, SD = 7.9) was greater than that of T2D (mean = 9.15, SD = 7.5, *p* < 0.01), with being ‘female’ and ‘parents of children and adolescents’ rendered as significant predictors of greater impact. Family members of T2D had a lower risk of experiencing a high family impact (FROM‐16 score >16) compared with T1D (RR 0.561, 95% CI 0.371–0.849).

**Conclusions:**

Compared to T2D, family members of T1D experience a greater impact on their QoL, particularly those caring for children and adolescents. These findings have clinical and resource implications, indicating a need to assess this impact as a part of routine diabetes care to support impacted family members. The FROM‐16 could assess this impact in routine practice and further facilitate referral of family members to appropriate support services.

## 1 INTRODUCTION

In the UK, over five million people are currently estimated to be living with diabetes, and this number is predicted to increase.^1^ There are, therefore, likely to be an equivalent number of families affected by diabetes. The pathogenesis of type 1 and 2 diabetes mellitus (T1D and T2D) is different, and T2D is more prevalent, comprising 90% of cases. However, in both types, family members play an important role in the management of diabetes,^2^, ^3^ which demands lifelong support in self‐management behaviours. Dealing with diabetes can be challenging for families, causing distress, imposing additional burdens or diminishing their quality of life (QoL).^4^, ^5^, ^6^, ^7^ Family members are even more concerned and distressed about diabetes than their relatives with diabetes.^8^, ^9^

Having a family member with diabetes can change the family dynamics, which then impacts the QoL of the individual family members and family relationships.^10^, ^11^ However, these influences on family dynamics might have positive results with better family cohesion and better problem‐solving. Conversely, there may be negative impacts of enhanced stress, anxiety, depression, anger, fear and helplessness, reducing family QoL.^12^, ^13^ This is because diabetes requires patients and families to adhere to new disciplines of multifaceted and complex treatment regimens, routine medication administration, regular clinic attendance, monitoring for symptoms, and other necessary lifestyle changes. Managing T1D is more challenging than managing T2D due to the more demanding insulin treatment and blood glucose monitoring. Therefore, dealing with diabetes daily can be challenging for families causing distress, impose additional burdens or diminish the quality of life.^14^, ^15^ It is important, therefore, that family members' emotional and psychological burden should be understood and addressed since family members play a crucial role in optimising diabetes management.^4^, ^10^ There is growing evidence that both patients' and family members' clinical and psychosocial outcomes can be improved when healthcare professionals undertake a family‐centred approach to chronic diseases, including in diabetes.^16^, ^17^, ^18^ One approach, which is feasible in time‐restricted healthcare settings, could be the use of the Family Reported Outcome Measure (FROM‐16)^19^ with a completion time of 2 min, alongside patient reported outcome measures (PROMS), thus ensuring holistic care.

The DAWN 2 (The second Diabetes Attitudes, Wishes and Needs) study, the biggest study on the family impact of diabetes conducted a decade ago with 2057 family members of people with diabetes (PWD) across 17 countries, revealed a substantial negative impact on family members.^2^ However, that study did not explore whether there were differences in the impact between family members of T1D and T2D. Since then, there have been advances in the treatment of diabetes, including statins therapy, innovative drugs, artificial intelligence and nanotechnology, possibly affecting the family impact of diabetes. Despite these advances in treatment, diabetes may continue over time to lead to complications: every week in the UK, diabetes leads to 184 amputations, more than 770 strokes, 590 heart attacks and 2300 cases of heart failure.^1^ Diabetes is a lifelong condition impacting both people with diabetes and their family members/partners, imposing a significant emotional, practical, and financial burden on patients and their families.^2^ Although new technologies can make diabetes management more efficient, people still require daily involvement from family members, including helping with device maintenance, monitoring blood glucose, encouraging lifestyle changes and ensuring adherence to treatment plans. It is important to understand this impact on family members and whether there is a difference in impact between T1D and T2D. This information is vital to inform resource allocation to provide targeted support to the impacted and high‐risk family members. There is currently a lack of scientific evidence on whether there is a difference in the family impact between T1D and T2D. This study, therefore, aims to measure the impact of T1D and T2D on the QoL of family members/partners of people with diabetes and assess if there is any difference in family impact. The hypothesis is that family members of PWT1D are more impacted by their relative's diabetes than family members of PWT2D, and this study will test this hypothesis.

## 2 METHODS

### 2.1 Study design and participant recruitment

The data used in this study came from a large online cross‐sectional study of family members/partners of people with a wide range of medical conditions.^21^ The study was carried out between April and November 2021 during the COVID‐19 pandemic. In this study, people with diabetes and their partners/family members were recruited online through Diabetes UK, Juvenile Diabetes Research Foundation (JDRF), Healthwise Wales (HWW) and Social Services Departments (SSDs) Wales. The study was open to UK family members/partners of PWD aged ≥18 years and capable of operating an electronic device. The family members/partners gave informed electronic consent after reading the participant information sheet embedded in the online questionnaire.

The main study outcome was the impact of a person's diabetes on the QoL of family members/partners, as measured by the FROM‐16. The predictors were type of diabetes, patient age, family member's relationship to the patient, gender and family member's occupation. Potential confounders were the patient's age, family members' occupation, relationship to the patient and family member's gender. The diagnosis of diabetes was self‐reported by patients and family members.

### 2.2 Assessment instrument

The impact on family members/partners was measured using the FROM‐16, a generic family QoL instrument, which measures the impact of any disease on the QoL of adult family members or partners, of patients of any age.^19^, ^20^ The FROM‐16 comprises 16 items, each with three response options: ‘Not at All’ (scoring 0), ‘A Little’ (scoring 1) and ‘A Lot’ (scoring 2). The 16 items are divided into two categories (domains): Emotional (comprising six items, maximum score of 12) and Personal and Social Life (comprising 10 items, maximum score of 20). The lowest possible score of the FROM‐16 is 0, and the highest is 32. The higher the total score, the greater the negative effect on the family member's QoL.

FROM‐16 was developed following interviews with 133 family members of patients across 26 medical specialities, exploring in depth impact of a relative's health condition, including diabetes, on family members.^19^ FROM‐16 has demonstrated high internal consistency (*n* = 120, Cronbach's *α* = 0.91) and high reproducibility (*n* = 51, ICC = 0.93), with a mean completion time of two minutes.^19^ The Cronbach's alpha calculated from the current diabetes study data was 0.92, confirming reliability of the FROM‐16 to measure family impact in diabetes. Confirmation of longitudinal validity of the FROM‐16 included data from 29 families affected by diabetes.^22^ Assessment of construct validity also included diabetes population and was proven through the correlation between FROM‐16 and WHOQOL‐BREF total scores (*n* = 119, *r* = −0.55, *p* < 0.001), and the correlation between FROM‐16 and the patient's overall health score (*n* = 120, *r* = −0.51, *p* < 0.001).^19^ The interpretation of scores is described using validated score meaning bands.^21^ Responsiveness to change has been established and the minimal important change (MIC) score value of FROM‐16 is 4.^22^ The FROM‐16 has been mapped to EQ‐5D‐3L^23^ for the potential use of inclusion of family impact of disease in health economic analysis.

### 2.3 Procedure

The online study was conducted using the Jisc academic survey platform15, which is compliant with the General Data Protection Regulation. The online study questionnaire had two sections; in Section 1, PWD completed some basic information (sex, age, occupation, health condition and country of residence) about themselves and chose their family member/partner to take part in the study. Section 2 was completed by the family member/partner of PWD and comprised some basic demographic questions (sex, age, occupation and relationship to patient) and FROM‐16.

The online questionnaire was available in two formats: patient and family member (FM) questionnaire or FM‐only questionnaire. In the FM‐only questionnaire, the family member completed patient demographic information.

Two patients and one family member were involved in the study as research partners who reviewed all study material.

As the study was conducted online during the COVID‐19 pandemic through patient support groups, only family members linked to support groups had a chance to participate in the study. However, to make participation more representative of the UK population, the study was open to all major national diabetes patient support groups. Other measures put in place to minimise bias included the creation of a database with edit checks ensuring a low probability of missing data thus bias.

### 2.4 Sampling strategy and sample size calculation

The study used non‐probability sampling with participants collected online through patient support groups. However, to reduce bias, the study encouraged all national diabetes support groups to participate to allow family members of PWD across all four regions of the UK to participate in the study.

The sample size was estimated using the following formula:

$$\text{Sample size}\;\left(n\right)=\frac{{Z^{2}}_{1-\alpha /2}P\;\left(1-P\right)}{E^{2}}\;=204.2.$$

The estimated sample size for this study was 204 participants, assuming a 5% significance level, a *z* value = 1.96 and a precision of 3.5%.

### 2.5 Ethics statement

Ethical approval was given by the Cardiff University School of Medicine Research Ethics Committee (SREC reference: 21/19).

### 2.6 Data analysis

Descriptive analysis was carried out and included calculating the mean, median, standard deviation and interquartile range of quantitative variables, as well as frequency and proportion for categorical variables. Non‐parametric tests, the Mann–Whitney *U* test and the Kruskal‐Wallis test were used to compare the impact between groups. Descriptive banding was assigned to the FROM‐16 scores to describe the severity of the impact on family members/partners across T1D and T2D. We determined the relative risk of high family impact using univariable and multivariable logistic regression with a log‐binomial link function. Covariates included type of diabetes (T1D and T2D), sex (female and male), age group (0–17, 18–29, 30–59, 60–75 and 76–96 years), occupation (unemployed, paid work or retired) and relationship status (spouse, parent, adult children and others). A full model including all the variables was fitted followed by a backward selection procedure to identify the optimal model. For the final multivariable model, fit statistics was assessed using the model providing the least fit statistics value. Confounding was controlled through multivariable regression modelling with age, sex, relationship to patient and occupation treated as covariates in the model. As a sensitivity measure, further analysis was conducted to better understand the data that is presented in the supplementary tables. Statistical analysis assumed a 5% significance level. Data were analysed using IBM SPSS Statistics for Windows, version 27, except for RR which was conducted using SAS Enterprise Guide 7.15.

## 3 RESULTS

A total of 261 family members/partners (mean age = 57.9 years, SD = 13.8; females = 68.2%) of PWD (mean age = 57.7, SD = 20.6; females = 38.3%; T1D =100; T2D = 161) completed the FROM‐16 (Table 1). Family members were mostly spouses/partners (67%), parents (14.6%) and sons and daughters (13.4%). Half of PWD (54.4%) and their family members (46%) were retired, while 24.5% of PWD and 37.2% of family members were in paid jobs. Most of the participants were from Wales (78.5%) followed by England (19.2%) (Table 1).

**TABLE 1:** Descriptive and sociodemographic characteristics.

Persons with diabetes (*n* = 261)		Mean (SD) or n (%)
Age (years)	Mean (SD)	57.7 (20.6)
	Median (IQR)	64 (72–50)
	Range	2–96
Gender	Male	159 (60.9)
	Female	100 (38.3)
	Prefer not to say	1 (0.4)
	Other	1 (0.4)
Occupation	In paid work	64 (24.5)
	Part‐time job	10 (3.8)
	Unemployed	6 (2.3)
	In unpaid work	1 (0.4)
	Education/training	12 (4.6)
	Homemaker	7 (2.7)
	Retired	142 (54.4)
	Rather not say	1 (0.4)
	Not applicable^a^	18 (6.9)
	England	50 (19.2)
	Northern Ireland	1 (0.4)
	Scotland	5 (1.9)
	Wales	205 (78.5)
Type of diabetes	Diabetes type 1	100 (38.30)
	Diabetes type 2	161 (161)
Family members of persons with diabetes (*n* = 261)		
Age (years)	Mean (SD)	57.9 (13.8)
	Median (IQR)	60 (68–49)
	Range	21–86
Gender	Male	81 (31)
	Female	178 (68.2)
	Prefer not to say	1 (0.4)
	Other	1 (0.4)
Occupation	In paid work	97 (37.2)
	Part‐time job	21 (8)
	Unemployed	4 (1.5)
	In unpaid work	1 (0.4)
	Education/training	4 (1.5)
	Homemaker	12 (4.6)
	Retired	120 (46)
	Rather not say	2 (0.8)
Relationship to patient	Spouse/partner	175 (67)
	Parent	38 (14.6)
	Adult child	35 (13.4)
	Other (sibling, father/mother in law, grandparent, uncle/aunt, grandson/granddaughter, brother/sister in law, nephew/niece, cousin, friend)	13 (4.9)

*Note*: ^a^Patients were children; hence, occupation was not applicable.

The FROM‐16 mean summary score for family members of PWD was 10.47 (SD = 7.8, median = 8, IQR = 12), with a mean score for emotional domain = 4.6 (SD = 3.3) and for personal and social domain = 5.9 (SD = 5) (Table 2). As for the individual FROM‐16 items, ‘being worried’ had the highest mean score of 1.2 (SD = 0.6), followed by ‘feeling frustrated’, ‘feeling sad’, ‘impact on family activities’, ‘effect on sleep’ and ‘sex life’ (Table 2). There was a significant difference in FROM‐16 total mean scores between male and female family members (*p* = 0.026), with females being impacted more by their relative's diabetes than males (Table S1). A significant difference between males and females was also noticed at the individual FROM‐16 item level, with females experiencing more impact across ‘being worried, feeling angry’, ‘feeling frustrated’, ‘difficulty caring’, ‘effect on eating habits’, ‘family relationships’ and ‘sleep’ due to their relative's diabetes (Table S1).

**TABLE 2:** Mean total FROM‐16 scores and individual item scores of family members/partners of people with diabetes (*n* = 261).

FROM‐16	Description	Mean (SD)	Median (IQR)	Range
Total FROM‐16 mean score	Overall	10.47 (7.8)	8 (12)	0–32
Domain score	Emotional domain	4.6 (3.3)	4 (5)	0–12
	Personal and social domain	5.9 (5.0)	4 (7)	0–20
FROM‐16 individual Items score	Worried	1.2 (0.6)	1 (1)	0–2
	Angry	0.4 (0.6)	0 (1)	0–2
	Sad	0.8 (0.7)	1 (1)	0–2
	Frustrated	0.9 (0.8)	1 (2)	0–2
	Talking about thoughts	0.7 (0.8)	0 (1)	0–2
	Difficulty caring	0.6 (0.7)	1 (1)	0–2
	Time for self	0.5 (0.7)	0 (1)	0–2
	Everyday travel	0.3 (0.6)	0 (0.5)	0–2
	Eating habits	0.6 (0.7)	1 (1)	0–2
	Family activities	0.8 (0.8)	1 (1)	0–2
	Holiday	0.7 (0.8)	0 (1)	0–2
	Sex life	0.8 (0.9)	0 (2)	0–2
	Work or study	0.3 (0.6)	0 (1)	0–2
	Family relationships	0.41 (0.6)	0 (1)	0–2
	Family expenses	0.6 (0.7)	0 (1)	0–2
	Sleep	0.8 (0.8)	1 (1)	0–2

### 3.1 Comparison between type 1 and type 2 diabetes

**FIGURE 1:**
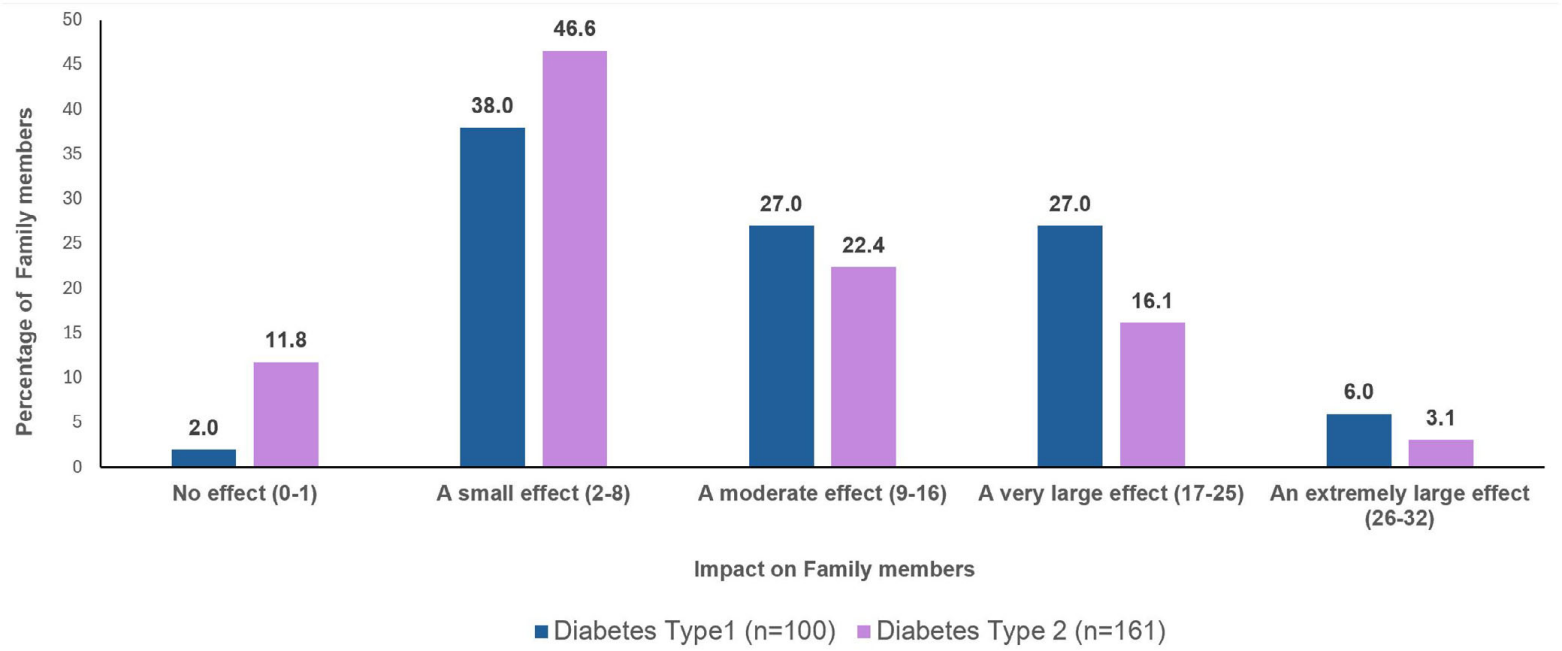
Impact on quality of life of family members/partners of people with diabetes type 1 and type 2.

Family members of PWT1D had a higher FROM‐16 summary score (mean = 12.61, SD = 7.9) than family members of PWT2D (mean = 9.15, SD = 7.5, *p* < 0.001). There was a significant difference between female family members of PWT1D and PWT2D (*p* = 0.001) with T1D female family members being more impacted, however, there was no significant difference between male family members of T1D and T2D (*p* = 0.090) (Table S2). The difference in impact between the family members of T1D and T2D was also noticed at the individual items level with T1D family members having significantly higher mean scores for feeling worried, feeling frustrated, talking about thoughts, having difficulty caring for their relative, effect on work or study, effect on holidays, effect on family relationships, effect on family expenses, effect on sleep (*p* < 0.01) and feeling angry (*p* < 0.05) (Figure 1).

**FIGURE 2:**
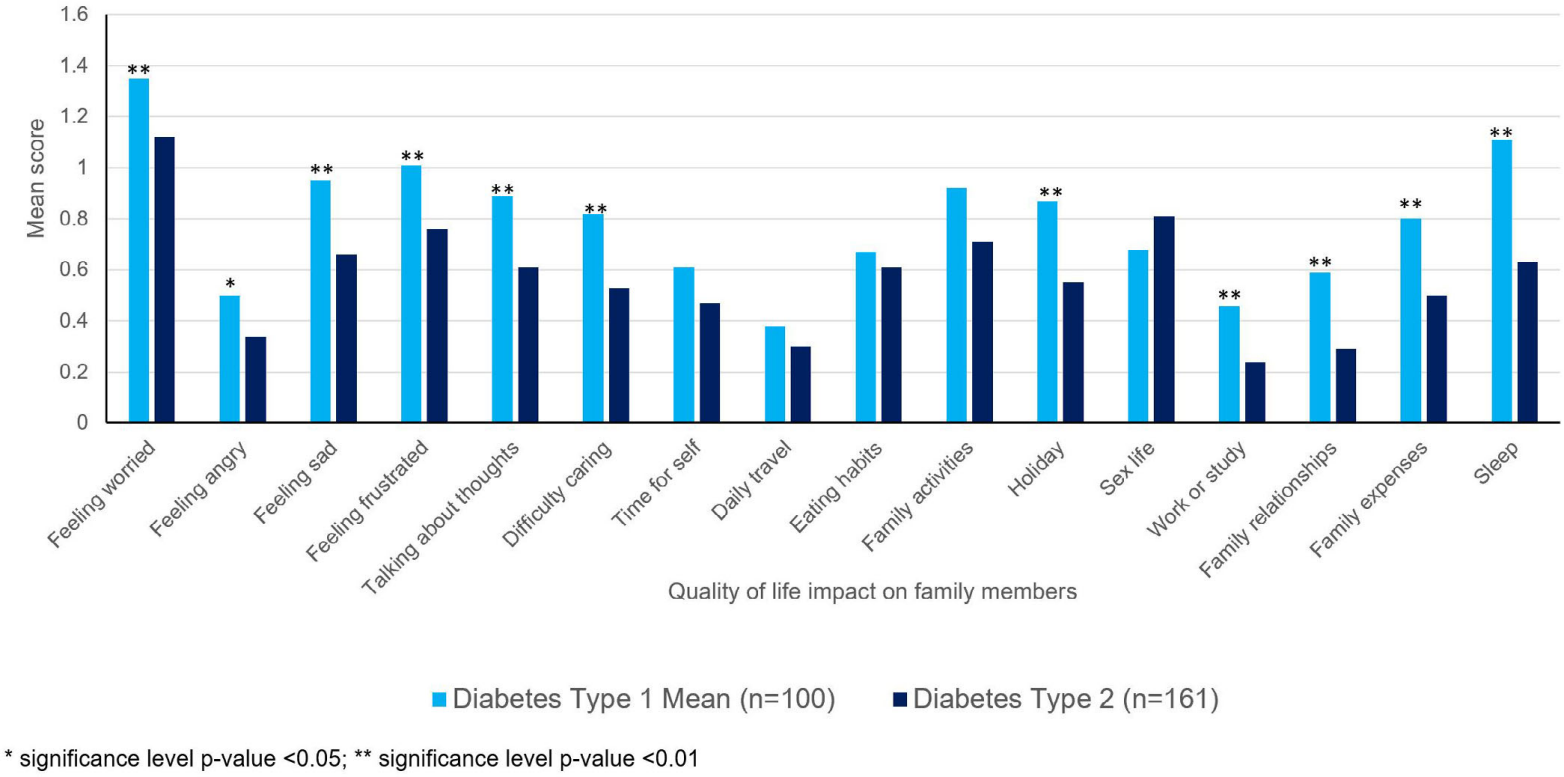
Family impact of diabetes type 1 and type 2 across individual FROM‐16 items.

The study also explored the degree of severity of impact experienced by the family members/partners^21^: 24.5% had a mean FROM‐16 score ≥17, indicating ‘a very large impact’ on the QoL of these family members. Only 8% of family members experienced ‘no impact’ (Table S3). Further analysis of the severity of impact indicated that family members of PWT1D (were more impacted) (33% having FROM‐16 ≥17) than the family members of PWT2D (19.2% FROM‐16 scores ≥17) (Figure 2).

### 3.2 Patient age and family impact

Family members of children and adolescents (age group 1 = 0–17 years) had the highest FROM‐16 scores (mean = 20.5, SD = 6.1), followed by family members of young people (age group 2 = 18–29 years). Family members of older adults experienced the least impact on their QoL, with the lowest FROM‐16 score (mean = 9.05). There was a significant difference between family members between age group 1 and age groups 2, 3, 4 and 5 (Table S4a,b). A scatter plot of the age of T1D and a family member's FROM‐16 total score showed a negative correlation indicating increasing QoL impact in family members with decreasing age of the person with diabetes (Figure S1).

### 3.3 Impact of a person's diabetes across relationships

The FROM‐16 mean total score differed depending on the family member's relationship with a person with diabetes. The QoL of parents (*n* = 33) was significantly impacted more (mean = 15.50, SD = 8.8, median = 17.5, range 0–28, IQR = 17.25) than that of spouses (mean = 9.17, SD = 6.8, median = 7, range 1–30, IQR = 10, *n* = 175; *p* < 0.01) (Tables S5a,b and S6a,b).

In the multivariable analysis, T2D family members had a lower risk of experiencing a high family impact compared with T1D family members (RR: 0.561, 95% CI 0.371–0.849) whereas females had a higher risk of experiencing a high family impact relative to males (RR 2.520, 95% CI 1.360–4.669) (Table 3).

**TABLE 3:** Factors associated with family impact of diabetes.

	Univariate				Multivariate			
Variables	Relative risk	Lower 95%	Upper 95%	*p* value	Relative risk	Lower 95%	Upper 95%	*p* value
Diabetes								
Type 2 diabetes versus type 1 diabetes	0.583	0.383	0.890	0.0123	0.561	0.371	0.849	0.006
FM Sex								
Female versus male	2.412	1.294	4.495	0.0056	2.520	1.360	4.669	0.003
Patient Age‐group (years)								
18–29 versus 0–17	0.482	0.244	0.951	0.0352	‐	‐	‐	‐
30–59 versus 0–17	0.253	0.147	0.437	<0.0001	‐	‐	‐	‐
60–75 versus 0–17	0.251	0.163	0.386	<0.0001	‐	‐	‐	‐
76–96 versus 0–17	0.234	0.109	0.502	0.0002	‐	‐	‐	‐
FM Occupation								
Paid work versus unemployed	0.368	0.245	0.552	<0.0001	‐	‐	‐	‐
Retired versus unemployed	0.210	0.127	0.348	<0.0001	‐	‐	‐	‐
FM Relationship								
Parent versus spouse	3.454	2.216	5.384	<0.0001	‐	‐	‐	‐
Adult child versus spouse	1.964	1.083	3.563	0.0263	‐	‐	‐	‐
Others versus spouse	1.923	0.795	4.652	0.1468	‐	‐	‐	‐

*Note*: Outcome variable: family impact with binary values of 0 and 1. Here, 1 represents a very large to extremely large family impact (FROM‐16 score >16) and 0 represents no to moderate impact (FROM‐16 score < 16). FM: family member; For occupation: unemployed includes unpaid work, education/training, homemaker, and paid work included paid work and part‐time work. For relationship, ‘others’ included sibling, father/mother in law, grandparent, uncle/aunt, grandson/granddaughter, brother/sister in law, nephew/niece, cousin, friend.

## 4 DISCUSSION

To our knowledge, this is the first study to explore the difference between the family impact of T1D and T2D. Although some studies have explored the impact of T1D or T2D on family members,^9^, ^24^, ^25^ there is a major evidence gap in the understanding of how impact on families varies with the type of diabetes. This information is important to inform allocation of resources to where the need is greatest. Our findings suggest that there is a significant difference in family impact between T1D and T2D, with T1D having more impact on family members, particularly when caring for children and adolescents.

Around one fourth of family members (24.5%) perceived a ‘very large’ or ‘extremely large’ negative impact of having a family member with diabetes on their QoL. These findings are consistent with those of Molla et al.,^26^ where 24.0% and 8.5% of the study participants reported moderate to severe and severe family burdens of diabetes mellitus. However, contrary to these findings, in the Dawn 2 study, only 8% of family members indicated a ‘poor’ or ‘very poor’ QoL impact of a person's diabetes.^2^

Although family members of PWD were impacted across all 16 items of FROM‐16, the most impacted aspects of life included being worried, frustrated, feeling sad, impact on family activities, sleep and sex life. These findings are consistent with the DAWN 2 study,^2^ where 61% were worried about the risk of hypoglycaemic events, 40% of family members experienced a high level of distress related to concerns about their relative with diabetes and 33.3% were frustrated about how best to help their relative with diabetes. In our study, 58.6% of family members experienced the impact on family activities. Kimbell et al.^7^ argue that caring for a young child with diabetes can have an impact on wider family life. For example, parents with T1D children may modify their own and/or their family's eating practices to manage mealtimes.^27^ Apart from the general overview of the impact of diabetes on family members, this study also explored how the family impact varies with the type of diabetes.

One third of family members of PW T1D experienced a ‘very large ‘or ‘extremely large’ effect on their QoL compared to 19% of family members of PWT2D. Family members of PWT2D had a lower risk of experiencing a high family impact compared to family members of PWT1D (RR: 0.561, 95% CI 0.371–0.849). This may be because T1D is managed using intensive insulin regimens, which involve multiple daily tasks (e.g. regular blood glucose monitoring, carbohydrate counting, calculating and administering insulin) and, therefore, may present more challenges for family members in everyday life compared to T2D, which is mainly controlled by oral hypoglycaemic drugs.

Although family members of T1D and T2D felt greatly worried and frustrated because of their relative's diabetes, family members of those with T1D were significantly more worried and frustrated. The high level of worry among family members of T1D could be attributed to concern about their relative's long‐term complications, severe hypoglycaemia^28^ and parents' concern about their child's future.^29^, ^30^

Compared to T2D, family members of T1D people experienced difficulty in sharing their thoughts and caring for their relatives. This is consistent with the literature where family members of T1D people reported difficulty coping, difficulty talking about their concerns, and needing more support from friends, family and healthcare providers.^28^, ^31^ Furthermore, family members of people with T1D experienced a greater impact on their sleep (mean = 1.11, min 0, max = 2) compared to family members of people with T2D (mean = 0.63, min =0, max = 2, *p* < 0.001). This is consistent with other studies where partners of T1D people reported disturbance to their sleep, with 44–55% waking up during the night to check on the person with diabetes or due to a diabetes‐related technology alarm.^32^, ^33^ Similarly, other studies have reported that parents' fear of their child's nighttime hypoglycaemia and consequent testing of blood glucose throughout the night, led to exhaustion and chronic sleep deprivation.^24^, ^34^

Family members of PWT1D reported a significant impact on holidays compared to family members of PWT2D, possibly requiring special arrangements for leisure activities and holidays to accommodate their needs with limited opportunities for spontaneity.^6^

Family members of PWT1D had a significant impact on family relationships. This is consistent with findings from other T1D studies.^6^, ^14^ Several studies highlighted how caregiving responsibilities impacted parents' lives and their relationship with their children.^6^, ^24^ In one study, mothers and fathers reported that their relationships changed as a result of constantly focusing on their child's diabetes.^7^ In our study, there was a significant impact of T1D on work (paid job) and study of family members compared to T2D. This is consistent with findings from other studies.^35^, ^36^, ^37^ Herbert et al.^37^ reported that 60% of parents (mostly mothers) with T1D children stated that having a child with diabetes influenced their employment decisions, with nearly one quarter reducing or quitting work. Harrington et al.^36^ reported that families with a child diagnosed with T1D experienced limitations in their workspace due to childcare.

In our study, family members of PWT1D experienced a significant increase in family expenses compared to family members of T2D. Katz et al.^26^ reported caring for a child with T1D was significantly more detrimental to parents' work and finances than caring for children with other or no special healthcare needs.^35^ The financial strain related to their child's diabetes care was also reported by parents who self‐identified as middle‐ to upper‐middle‐class.^30^

Several studies have found that the greater the age of the person with T1D, the lower the effect of diabetes on the family.^5^, ^25^ Consistent with these findings, there was a negative correlation between the age of a person with T1D and the total FROM‐16 score in our study. Furthermore, female family members were three times more likely to be impacted than males, which is consistent with other studies.^38^, ^39^, ^40^, ^41^ However, of specific interest, female family members of T1D were significantly more affected than female family members of T2D.

Family impact of overall diabetes was experienced in all relationships (spouse/partner, parent, adult child, others) with T1D having significantly more impact than T2D. However, parents of T1D were significantly more impacted than spouses/partners. This could be possibly due to the amount of care and vigilance needed to manage their child's diabetes, as young children may not be capable of comprehending or managing their condition independently.^42^

Our findings are consistent with studies from other countries. For example, a large US study by Harrington et al.^36^ on T1D showed that ‘worry,’ ‘feeling upset’, ‘impact on sleep’ and ‘work’ were the most frequently reported impacts of diabetes on parents. These results resonate with our findings. A study by Awadalla et al.^14^ in the Kingdom of Saudia Arabia showed that T1D caregivers had significantly lower QoL scores than T2D caregivers and the general population. Consistent with our findings, an Iranian study by Ghorbani et al.^43^ reported high levels of stress and negative emotions in family caregivers of patients with T1D, leading to lower caregiver QoL.

### 4.1 Comparison with other FROM‐16 studies

A study on the impact on family members of people with chronic diseases across 26 medical specialities (FROM‐16 mean = 12.4)^38^ and another on family members/partners of oncology patients (FROM‐16 mean = 11.8)^44^ reported lower FROM‐16 mean scores than that of the family impact of T1D in this study (FROM‐16 mean = 12.61) but higher scores than that of T2D (FROM‐16 mean = 9.15). A global study on the family impact of COVID‐19 survivors (FROM‐16 mean = 15.0)^40^ and a global study, also carried out during the pandemic, on myalgic encephalomyelitis/chronic fatigue syndrome (mean = 17.9),^45^ both reported higher impact on family members than found in our diabetes study (overall mean = 10.47). However our subgroup of family members of children and adolescents with T1D experienced a greater impact (mean = 20).

Our study has some limitations. As the study was conducted during the COVID‐19 pandemic, this may have influenced the results. However, the study findings are consistent with earlier findings from other diabetes studies conducted before and after the pandemic.^2^, ^37^ The study has the potential for selection bias as it was conducted online with patient support groups. This may have resulted in the participation of only those family members who were registered with patient support groups and who could use electronic devices, limiting the diversity of the sample and potentially affecting the generalizability of the results. Although the study used a validated instrument, the FROM‐16, to measure the impact of diabetes on family members and partners, the outcome is based on self‐reported data, which may have introduced reporting bias.

The study did not ask questions about ethnicity, socioeconomic status or cultural background; therefore, we cannot comment on the diversity of the sample. The absence of information on these background data about participants restricts understanding concerning how these differences may affect a person's diabetes. For future studies, it would be interesting to assess data stratified by income, locality of care and educational level of parents of children with T1DM. Furthermore, future studies should consider using objective measures to enhance data reliability. Future studies should incorporate measures of glycemic control, diabetes severity and complications and explore their relationship with family impact. This was not possible in the context of our online study. Additionally, future studies should employ a mixed‐method approach to triangulate findings and provide richer insights.

The study does not explore the long‐term effects of diabetes on family QoL or the impact of other health conditions that family members might face. In future, longitudinal studies should be carried out to assess the long‐term impact of diabetes and associated comorbidities on families. This could be by repeated use of FROM‐16 and by using the Major Life Changing Decision Profile (MLCDP).^46^

The study design did not deliberatively discriminate between different regions of the UK, though subjects were predominantly from Wales. Because of the anonymous nature of the subject self‐selection, it is not possible to be certain of the generalisability of the results to all regions of the UK. Although diabetes care could be different across the different healthcare systems and the details of family impact of diabetes might vary across different countries, a key message of our study is that there is likely to be an important family impact of diabetes worldwide.

Despite some limitations, this study provides valuable insights into the differential impact of T1D and T2D on family members' QoL, with important clinical implications. Although the overall FROM‐16 mean score in this study pointed to a moderate impact of diabetes on family members, one‐quarter of family members experienced a very large to extremely large impact on their QoL. Consistent with findings from other studies,^2^, ^9^, ^32^, ^33^, ^36^ family members in our study experienced a high emotional burden, impact on family activities, sex life and sleep. The study also showed that family members of PWT1D are more impacted than PWT2D, particularly parents of young children and adolescents. Compared to T2D family members, T1D family members experienced significant impact across the following FROM‐16 items: feeling worried, sad and frustrated, difficulty in caring for loved ones and sharing thoughts, work/studies, impact on holidays, family relationships, family expenses and sleep.

### 4.2 Implications for practice

The findings of our study suggest that family members of people with T1D are impacted more compared to family members of people with T2D, with parents of children and adolescents being significantly impacted more. The healthcare providers working with T1D families and, particularly, families of children and adolescents with T1D should take into consideration the impact of caring on families and the challenges they face in managing a person's diabetes. There is a need to measure this impact in routine clinical practice alongside PROMs as well as the inclusion of the patient and their family member in the multi‐disciplinary team meetings to understand and respond to the needs of family members. Responses might for example, include arranging a further assessment with a psychologist or social worker as part of a diabetes clinic appointment to provide needed support and care to impacted family members. This, in turn, could improve the patient's diabetic outcomes and reduce the financial burden of providing long‐term care for people with diabetes.

Family impact is operationalised through use of family‐reported outcome measures (FROMs), which are family quality of life instruments^47^ and through burden scales such as the Zarit Burden scale.^48^ Although over the last three decades, there has been growing interest in measuring the impact of disease on family members and in the support of those affected, the use of these instruments is still largely restricted to research.^47^ There is a need to use FROMs in regular clinical practice alongside PROMs to inform holistic medical care. Additionally, the measurement of family impact is important in value‐based healthcare (VBHC), a new paradigm for allocating healthcare resources that is increasingly being embraced worldwide.^49^, ^50^ One of the important components included in VBHC is societal value, a key element of which is to measure the impact of a condition (and the gains from treating or controlling the condition) on a person's family. The FROM‐16, the generic validated family QoL measure, with score meaning bands, could be used in routine clinical practice to understand the support needs of families of people with diabetes. The FROM‐16 measures the negative impact of having a family member with a health condition and comprises 16 aspects of family impact that have a direct bearing on physical, social and psychological wellbeing. While FROM‐16 includes impact on family activities and family relationships, it does not measure resilience. If healthcare professionals wish to measure aspects beyond QoL, such as family functioning or resilience, the use of other additional instruments such as ‘Family Resilience Assessment Instrument and Tool’ (FRAIT)^51^ can be considered.

## 5 CONCLUSIONS

This study demonstrates that family members of people with T1D, and in particular parents of children and adolescents with T1D, experience a great impact of caring on their QoL. There is a need to measure this impact in routine practice to provide tailored support to prevent burnout and further complications for impacted family members as a part of holistic medical practice.

## AUTHOR CONTRIBUTIONS

RS primarily carried out the study including data collection, analysis and interpretation, wrote the first draft and revised all documentation. SS and AYF equally contributed to the design, interpretation and supervision of the study and revised all study documentation and the manuscript. JRI, KO and SJN provided advice during the study and helped revise study documentation. SJN reviewed patient and family member documents. AYF, SS, KO, JRI, FMA SJN, LME and LG reviewed the manuscript and agreed on the final submitted version of the manuscript.

## FUNDING INFORMATION

There was no external funding for this Cardiff University study.

### PEER REVIEW

The peer review history for this article is available at https://www.webofscience.com/api/gateway/wos/peer-review/10.1111/dom.16058.

### 1 CONFLICTS OF INTERESTS STATEMENT

RS, KO, LG, LME and SJN declared no competing interest; FMA is employed by Cardiff University, which generates income from the use of FROM‐16; AYA reports personal fees from Novartis Lecture honorarium, personal fees from Medscape Podcast honorarium, personal fees from Eli Lilly Lecture honorarium, outside the submitted work; AYF and SS are joint copyright owners of the FROM‐16: Cardiff University receives royalties for some uses, they receive a share under standard university policy; JRI receives a stipend as Editor‐in‐Chief of the British Journal of Dermatology and an authorship honorarium from UpToDate. He is a consultant for Abbvie, Boehringer Ingelheim, ChemoCentryx, Citryll, Insmed, Kymera Therapeutics, MoonLake, Novartis, UCB Pharma, UNION Therapeutics, and Viela Bio., all in the field of hidradenitis suppurativa (HS). He is co‐copyright holder of HiSQOL, Investigator Global Assessment and Patient Global Assessment instruments for HS. His department receives income from copyright of the Dermatology Life Quality Instrument (DLQI), Family Reported Outcome Measure (FROM‐16) and related instruments.

## ETHICS STATEMENT

The study was approved by the Cardiff University School of Medicine Research Ethics Committee (SREC reference: 21/19).

## CONSENT TO PARTICIPATE

All patients and family members gave their electronic informed consent.

## Supporting information


**Data S1.** Supporting Information.

## Data Availability

The data are available from the authors on reasonable request according to Cardiff University regulations.
