# Supplementary material for: Comparison of the impact of type 1 and type 2 diabetes on quality of life of families of patients: A UK cross‐sectional study
Source: Diabetes Obes Metab. 2024 Nov 24;27(2):652–62. doi: 10.1111/dom.16058 (PMC11701181; doi:10.1111/dom.16058)

Supplementary material

Comparison of the impact of Type 1 and Type 2 Diabetes on Quality of Life of Families of Patients: A UK Cross-Sectional Study

INDEX: Tables/figures

Table S1 Comparisons[†] of family impact of person's diabetes based on family members' gender[¥] (n=261)

Table S2 Comparison between family impact of Diabetes Type 1 and 2

Table S3 FROM-16 score banding describing the impact on quality of life of family members/partners of people with diabetes[20] (n=261)

Table S4a FROM-16 scores across different age groups

Table S4b Pairwise comparisons[T] of family impact across age groups

Table S5a Overall FROM-16 mean score for family members of diabetic patients

Table S5b Pairwise comparison[†] of QoL impact across relationships (spouse/partners, adult children, parents, siblings and other relatives of people with Diabetes)

Table S6a Comparison[†] of impact on QoL across relationships FROM-16 score for Diabetes type 1 and type 2 across family member's relationship to patients

Table S6b Pairwise comparisons[T] of family impact across family member relationships to patients within Type 1

Figure S1a Scatter plot of total FROM-16 score by patient age for Type 1 Diabetes

Figure S1b Scatter plot of total FROM-16 score by patient age for Type 2 Diabetes

Table S1 Comparisons† of family impact of person's diabetes based on family members' gender¥ (n=261)

FROM-16	Mean score		p-value
	Male (n=81)	Female (n=178)	
**Total FROM-16**	8.69	11.24	0.026*
**FROM-16 Domain**			
Emotional	3.78	4.95	0.010**
Personal and Social	4.91	6.29	0.128
**FROM-16 items**			
Worried	1.06	1.28	0.013*
Angry	0.27	0.45	0.008**
Sad	0.7	0.8	0.295
Frustrated	0.7	0.92	0.040*
Talking about thoughts	0.59	0.77	0.095
Difficulty caring	0.44	0.74	0.002**
Time for self	0.37	0.6	0.074
Everyday travel	0.31	0.34	0.902
Eating habits	0.52	0.69	0.047*
Family activities	0.72	0.82	0.300
Holiday	0.62	0.69	0.595
Sex life	0.7	0.79	0.438
Work or study	0.21	0.37	0.073
Family relationships	0.21	0.49	0.001**
Family expenses	0.54	0.65	0.319
Sleep	0.72	0.85	0.225

† Mann Whitney U test; *p ≤ 0.05, **p ≤ 0.01, 2-tailed. (p values were calculated using mean rank scores but mean scores are presented here for ease of understanding);¥one family member identified as 'other' had FROM-16 score =7 and one other family member did not want mention gender had FROM-16 score=23

Table S2 Comparison between family impact of Diabetes Type 1 and 2

Mean FROM-16 score	Diabetes Type 1	Diabetes Type 2	p-value**
Total FROM-16 (n=261)	12.61 (7.9)	9.15 (7.5)	<0.001
Emotional domain	5.52 (3.3)	4.02 (3.1)	<0.001
Personal & Social life domain	7.09 (5.3)	5.12 (5.0)	0.001
Male (n=81)	9.61 (8.5)	8.12 (7.3)	0.090
Female (n=178)	13.9 (8.5)	9.61 (7.5)	0.001

Note: † Mann Whitney U test; **p ≤ 0.01, 2-tailed. (p values were calculated using mean rank scores but mean scores are presented here for ease of understanding)

Table S3. FROM-16 score banding describing the impact on quality of life of family members/partners of people with diabetes (n=261)[20]

FROM-16 score banding	Number of family members	% of family members
No effect (0-1)	21	8.0
A little effect (2-8)	113	43.3
A moderate effect (9-16)	63	24.1
A very large effect (17-25)	53	20.3
An extremely large effect (26-32)	11	4.2
**Total**	**261**	**100**

Table S4a FROM-16 scores across different age groups

Family member	FROM-16				Domain score	
(Age group)	Mean(SD)	Median	Range	IQR	Emotional	Personal and Social life
Group 1 (0-17 yrs) n=18	20 (6.1)	20.5	7-27	8.25	7.78	12.22
Group 2 (18-29 yrs) n=16	13.2 (8.8)	10.0	3-28	14.00	6.56	6.63
Group 3 (30-59 yrs) n=66	10.1 (6.5)	9.0	0-23	11.25	4.56	5.50
Group 4 (60-75 yrs) n=128	9.05 (8.2)	6.0	0-30	12.00	3.88	5.18
Group 5 (76-96 yrs) n=33	10.30 (8.2)	7.0	1-32	11.50	4.79	5.52

Table S4b Pairwise comparisons [T] of family impact across age groups

Sample 1-Sample 2	Test Statistic	Std. Error	Std. Test Statistic	p-value	Adj. Sig.[a]
Age group 4-Age group 5	-9.97	14.72	-0.68	0.498	1
Age group 4-Age group 3	14.62	11.43	1.28	0.201	1
Age group 4-Age group 2	39.12	19.99	1.96	**0.05**	0.504
Age group 4-Age group 1	99.02	18.98	5.22	**0.001**	0
Age group 5-Age group 3	4.64	16.07	0.29	0.773	1
Age group 5-Age group 2	29.15	22.97	1.27	0.204	1
Age group 5-Age group 1	89.05	22.09	4.03	**0.001**	**0.001**
Age group 3-Age group 2	24.51	21.01	1.17	0.243	1
Age group 3-Age group 1	84.40	20.05	4.21	**0.001**	0
Age group 2-Age group 1	59.90	25.9	2.312	**0.021**	0.208

[T]Kruskal-Wallis-I way ANOVA Test.Each row tests the null hypothesis that the Sample 1 and Sample 2 distributions are the same. Asymptotic significances (2-sided tests) are displayed. The significance level is 0.050.[a] Significance values have been adjusted by the Bonferroni correction for multiple tests.

Table S5a Overall FROM-16 mean score for family members of diabetic patients

Relationship of family member to a person with diabetes	Overall FROM-16			
	Mean(SD)	Median	Range	IQR
Spouses/partners (n=175)	9.17 (6.8)	7.0	0-30	10
Parents (n=38)	15.50 (8.8)	17.5	0-28	17.25
Adult children (n=35)	11.51 (8.4)	9.0	0-32	14
Siblings (n=7)	9.43 (8.7)	5.0	1-21	19
Others (n=6)	11.83 (11.0)	9.0	1-29	19.75

Table S5b Pairwise Comparisons[T] of QoL impact across relationships (spouse/partners, adult children, parents, siblings and other relatives of people with Diabetes)

Sample 1-Sample 2	Test Statistic	Std. Error	Std. Test Statistic	Sig.	Adj. Sig.[a]
Spouse-Others	-3.702	21.674	-.171	.864	1.000
Spouse-Adult child	-18.777	13.960	-1.345	.179	1.000
Spouse-parent	-53.891	13.493	-3.994	<0.0001	<0.0001
Others-Adult child	15.075	24.488	.616	.538	1.000
Others-parent	50.188	24.225	2.072	.038	.230
Adult child-parent	35.114	17.664	1.988	.047	.281

[T]Kruskal-Wallis-I way ANOVA Test. Each row tests the null hypothesis that the Sample 1 and Sample 2 distributions are the same.

Asymptotic significances (2-sided tests) are displayed. The significance level is assumed when the p-value <0.05. [a]Significance values have been adjusted by the Bonferroni correction for multiple tests.

Table S6a Comparison[†] of FROM-16 score for Diabetes type 1 and type 2 across family member's relationship to patients

**Relationship of family member to a person with diabetes**	**Diabetes Type 1 Mean (SD)**	**Diabetes Type 2 Mean (SD)**	**p-value[†]**
Spouses/partners (n=175)	10.04 (6.7)	8.83 (6.9)	0.218
Parents (n=38)	17.21(8.0)	4.20 (4.5)	0.002
Adult children (n=35)	10.67 (6.7)	11.96 (9.3)	0.972
Siblings (n=7)	12.00 (9.0)	3.00 (2.8)	0.241
Others (n=6)	13.00	11.60 (12.3)	0.770

[†] Mann Whitney U test; *p ≤ 0.05, **p ≤ 0.01, 2-tailed. (p values were calculated using mean rank scores but mean scores are presented here for ease of understanding); Diabetes Type 1 (Spouse /Partner=49, Parent=33, Adult children=12, Sibling=5, Other=1); Diabetes Type 2 Spouse/Partner=126, Adult children=23, Parent=5, Sibling=2, Other=5)

Table S6b Pairwise comparisons[T] of family impact across family member relationships to patients within Type 1

Sample 1-Sample 2	Test Statistic	Std. Error	Std. Test Statistic	p-value*	Adj. p-value[a]
Sibling- Spouse/Partner	3.666	29.061	0.126	0.900	1.000
Sibling-Others	-15.964	41.946	-0.381	0.704	1.000
Sibling-Son / Daughter	22.443	31.217	0.719	0.472	1.000
Sibling- Parent	57.556	31.011	1.856	0.063	.634
Spouse/Partner-Others	-12.299	31.303	-0.393	0.694	1.000
Spouse/Partner-Son/Daughter	-18.777	13.960	-1.345	0.179	1.000
Spouse/Partner- Parent	-53.891	13.493	-3.994	**0.001**	0.001
Others-Son/Daughter	6.479	33.314	0.194	0.846	1.000
Others- Parent	41.592	33.121	1.256	0.209	1.000
Son/Daughter- Parent	-35.114	17.664	-1.988	0.047	0.468

[T]Kruskal-Wallis-I way ANOVA Test
Each row tests the null hypothesis that the Sample 1 and Sample 2 distributions are the same. Asymptotic significances (2-sided tests) are displayed.
*The significance level is 0.050. [a.] Significance values have been adjusted by the Bonferroni correction for multiple tests.

Figure S1a Scatter plot of total FROM-16 score by patient age for Type 1 Diabetes

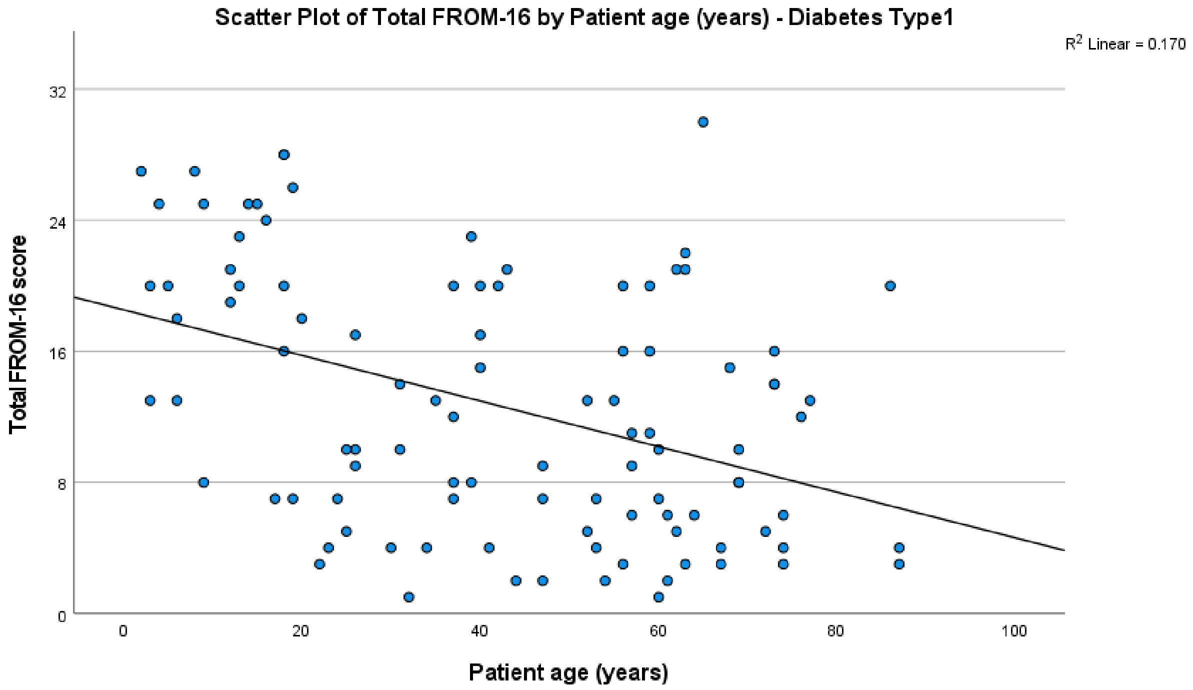

S1b Scatter plot of total FROM-16 score by patient age for Type 2 Diabetes

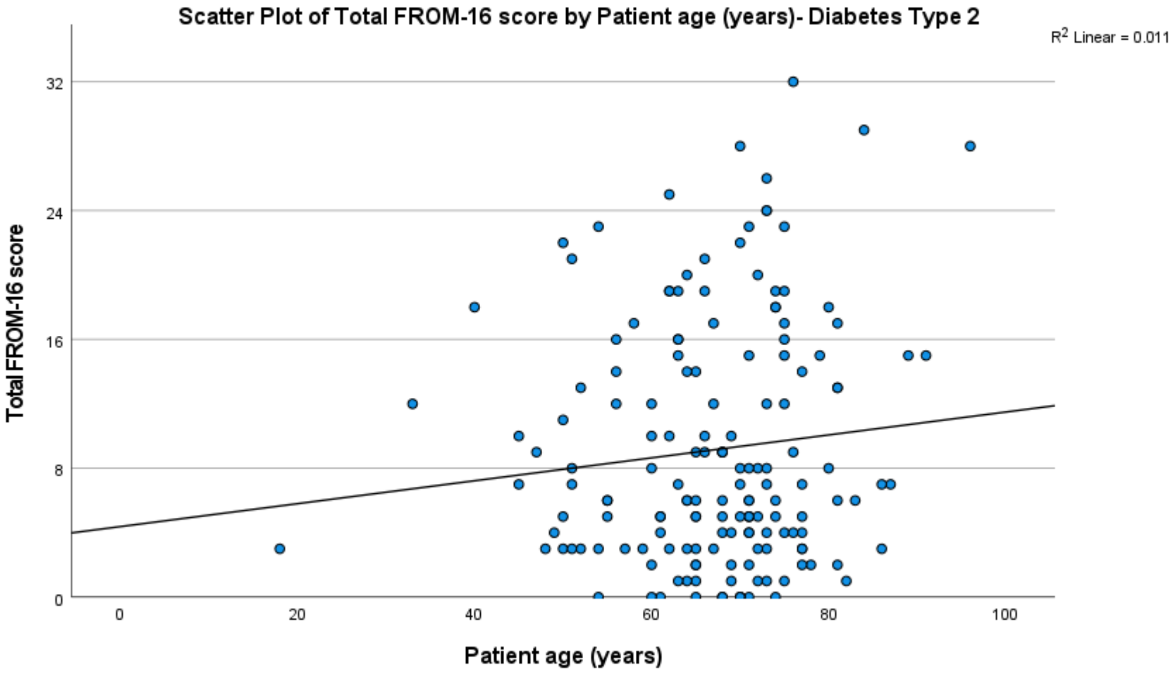

Supplement: Supplementary file 1 — Data S1. Supporting Information. [file DOM-27-652-s001.pdf]
